# Polyaxial locking plates in treating distal humeral fractures: a comparative randomized trial for clinical outcome

**DOI:** 10.1186/s12891-017-1910-9

**Published:** 2017-12-28

**Authors:** Moritz Crönlein, Martin Lucke, Marc Beirer, Dominik Pförringer, Chlodwig Kirchhoff, Peter Biberthaler, Karl F. Braun, Sebastian Siebenlist

**Affiliations:** 1 Department of Trauma Surgery, Klinikum rechts der Isar, Technical University of Munich, Ismaninger Strasse 22, 81675 Munich, Germany; 2 Department of Orthopaedic Sports Medicine, Klinikum rechts der Isar, Technical University of Munich, Ismaninger Strasse 22, 81675 Munich, Germany; 3 Department of Trauma Surgery, Chirurgisches Klinikum München Süd, Am Isarkanal 30, 81379 Munich, Germany

**Keywords:** Distal humeral fracture, Locking plate, Anatomical preshaped, Polyaxial, Outcome

## Abstract

**Background:**

Management of distal humeral fractures remains to be one of the most challenging aspects in trauma surgery. Low profile plating systems with variable angle screw fixation represent a crucial advancement to the established angular stable locking plates with considerable attention in current research. The aim of the prospective randomized trial was to review the preliminary results and patients’ outcome following treatment with these newly developed implants and to rule out potential differences in fracture treatment of two different plating systems.

**Methods:**

Twenty patients with distal humeral fractures (AO 13-A1 – AO 13-C3) were included in the current study since 2014. After completing the randomization plan, patients were distributed into two groups for different variable angle locking plates (DePuy Synthes® VA-LCP vs. Medartis**®** Aptus Elbow). Functional elbow scoring (ROM, MEPS, QuickDASH) served as primary outcome parameter, while radiological fracture consolidation served as secondary outcome parameter. Follow-ups were conducted 6 weeks, 12 weeks, 6 months and 12 months after the operation.

**Results:**

Seventeen of 20 patients (85%) concluded all follow-up examinations. Postoperative elbow extension deficiencies showed significant differences between the two groups in all follow-up examinations with a mean of Ø 18 +/− 7.4 degrees in the DePuy Synthes® VA-LCP group compared to a mean of Ø 6.5 +/− 7.5 degrees in the Medartis**®** Aptus Elbow group (*p* = 0.002) 12 months postoperatively. Functional scoring showed a disparate pattern. The Medartis**®** Aptus Elbow group achieved significantly better MEP scores during follow-up. However, the analysis of the QuickDash revealed better results of the DePuy Synthes® VA-LCP group in the first half and better results of the Medartis**®** Aptus Elbow group in the second half of the follow-up examination instead.

**Conclusions:**

Considering the complexity of distal humeral fractures, the usage of anatomically preshaped low profile variable angle locking plates for operative treatment leads to good clinical results. Even though there might be some advances of the Medartis**®** Aptus Elbow plating system concerning postoperative ROM and elbow function, a consistent difference in the overall clinical outcome between the two plating systems could not be detected.

**Trial registration:**

https://clinicaltrials.gov/ct2/show/NCT03272490 Retrospectively Registered 1. September 2017.

## Background

Distal humeral fractures are still very rare accounting for only 1–2% of all fractures [1]. Concerning the complex fracture pattern of these fractures together with the growing age of the patients, management remains to be one of the most challenging aspects in modern trauma surgery [2]. Dealing with these challenges, a large variety of different treatment options can be found in current literature, ranging from conservative treatment [3, 4] to different operative fixation techniques [5–8] up to total joint replacements [9].

The essential goal to achieve best results for patients suffering from distal humeral fractures is to restore joint congruency irrespective of patient’s age. Considering that, conservative treatment frequently leads to a painful limited range of motion (ROM) quite often. Over the last decades, therefore, open reduction and internal fixation (ORIF) is known to be the treatment of choice [10]. Due to the importance of the distal humerus for elbow function and stability, new specific angular stable implant systems and innovative fixation techniques have been developed in the recent past [11]. Biomechanical analysis of angle stable locking plates already show promising results for fracture treatment especially in the osteoporotic bone [12]. As an advancement to the known angle stable locking plates where screws can only be placed in the predetermined direction, low profile plating systems with a variable angle for screw fixation gain considerable attention in current research. The usage of anatomically preshaped plates with variable possibilities for angular stable screw fixation already showed promising results in dealing with various complex fracture types like distal femur fractures [13], distal tibial fractures [14], distal radial fractures [15], clavicle fractures [16] or radial head fractures [15, 17]. However, little about these modern implants is known in terms of dealing with distal humeral fractures.

With the Medartis**®** Aptus Elbow distal Humerus Plates 2.8 (Medartis®, Basel, Switzerland) and the DePuy Synthes® VA-LCP distal Humerus Plates 2.7/3.5 (DePuy Synthes®, Umkirch, Germany), two modern plating systems have recently been introduced. Both plating systems consist of an anatomically preshaped low-profile design with variable angle for locking screw fixation and especially designed for fracture treatment of the distal humerus. Therefore, the aim of the present prospective randomized trial was to review the preliminary functional outcomes following treatment with these anatomically preshaped low-profile plating implants in the short-term and to rule out potential differences between both plating systems.

## Methods

### Patients

Before study initiation the approval of the local ethics committee of (Trial Number 253/14) was obtained. The comparative prospective randomized clinical trial included 20 patients with fractures of the distal humerus recruited from a level-one university trauma centre. Patient recruitment was conducted between 03/2014 and 12/2015. Fractures of the distal humerus, as defined by the AO classification system (AO 13-A1 – AO 13-C3), were included in this study as they were identified by the treating surgeon being applicable for locking plate treatment and after written informed consent of the patients was obtained. All patients were randomized following a randomization plan (Randlist®, DatInf GmbH, Tübingen, Germany) for either being treated with the DePuy Synthes® VA-LCP 2.7/3.5 mm (DePuy Synthes®, Umkirch, Germany) or with the Medartis**®** Aptus Elbow system 2.0/3.8 mm (Medartis®, Basel, Switzerland). Both include anatomically preshaped, polyaxial angular stable locking plates in various sizes. Angle stable screw positioning with the freedom of +/− 15° off-axis screw placement gives the surgeon a large variety in both systems.

The inclusion criteria involve all patients from the age of 18 to 95 years who suffered from a distal humeral fracture (AO 13-A1 – AO 13-C3) that had to undergo operative treatment. The exclusion criteria involve all under-aged patients (< 18 years), pregnant patients and patients with a mental disorder as well as patients under comprehensive legal support. In addition, pathological fractures had been excluded from the study.

### Surgical technique

All patients were operated by consultant orthopaedic and trauma surgeons, experienced in upper extremity surgery. The mean interval between injury and operation was 2.4 days (range 0–12 days). General anaesthesia was used in all cases and a single dose of 1.5 mg cephalosporin was given preoperatively for prophylaxis. Patients were positioned in prone position with the injured arm on a radiolucent, small padded arm holder. Under tourniquet control, the posterior approach (Bryan-Morrey) to the distal humerus was performed in all cases. Additional olecranon osteotomy was performed in 7 cases (3× DePuy Synthes® vs. 4× Medartis**®** Aptus Elbow) presenting with AO type 13 C2 and AO type 13 C3 fractures. Postoperatively, physiotherapy was initiated using the same rehabilitation protocol for both groups. Passive and active assisted ROM was permitted immediately without limitations, while weight bearing was restricted for 6 weeks.

### Follow-up evaluation

All patients were initially followed-up 6 weeks after operation. Additional follow-ups were performed 3, 6 and 12 months postoperatively. The follow-up examinations were carried out by an independent investigator not involved in patient’s initial surgical treatment (MC) in the outpatient clinic of our level-one university trauma centre.

For the assessment of pain, the visual analogue scale (VAS), ranging from 0 “no pain” to 10 “worst imaginable pain” was documented. ROM and collateral ligament stability were registered on standardized scoring sheets. For subjective evaluation, patients rated their satisfaction for elbow use on a scale of 1 to 6 (1–highly satisfied; 2–satisfied; 3–moderate; 4–sufficient; 5–unsatisfied; 6-very unsatisfied). Moreover, sensomotoric disturbances and postoperative complications were recorded. Minor complications had been defined as complications that could be treated conservatively (e.g. superficial wound infections, delayed union etc.), whereas major complications needed operative revision (e.g. secondary loss of reduction, non-unions, severe wound infections etc.). For functional upper extremity and elbow scoring the shortened Disabilities of the Arm, Shoulder and Hand Score (QuickDASH) and the Mayo Elbow Performance Score (MEPS) were comprised. Postoperative x-rays were evaluated with special respect to bony healing, secondary loss of reduction and heterotopic ossifications.

### Statistics

Statistical analyses were performed using the statistical software SigmaStat (version 3.5; Systat Software, San Jose, CA, USA). The scores at certain time points were compared with an independent t test after a normality check had been passed and equal variances had been detected. Normal distributed data with unequal variances would have been compared with Welch’s t test. Arbitrarily data was tested with Mann-Whitney U test. The significance level was set at *p* = 0.05.

## Results

### Epidemiological data

**Fig. 1:**
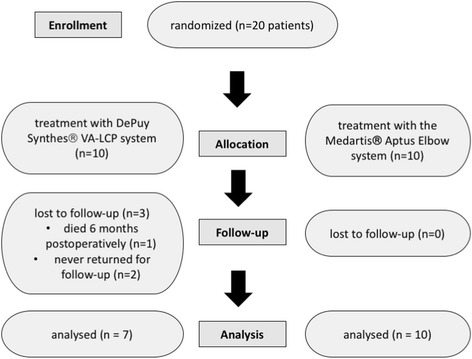
Flow diagram of the progress through the phase of the prospective randomized trial

Seventeen of 20 patients (85%) were able to conclude all follow up examinations (see Fig. 1). Three patients were lost to follow-up. One patient died 6 months postoperatively of unrelated cause to the surgery, the other two patients never returned for postoperative clinical and radiological control of unknown cause. These both patients also couldn’t be found for follow-up survey. The remaining 17 patients presented with a mean age of 79 years (range 31–91 years) at the time of injury with no statistically differences between groups. Most common injury types were falls from less than three meters. Gender distribution showed differences with only 1 male patient compared to 16 female patients (see Table 1).

**Table 1:** A total of 17 patients, presenting with a distal humeral fracture (AO-type 13 A-C), were recruited to this prospective randomized trial, either being treated with DePuy Synthes® VA-LCP or Medartis**®** Aptus Elbow plating systems. There were no statistically significant differences in patients` demographics or injury characteristics between the groups

Characteristics	DePuy Synthes® VA-LCP	Medartis**®** Aptus Elbow
Mean age (years)	81	76
Sex (male: female)	0:7	1:9
AO classification		
13-A	2	1
13-B	0	0
13-C	5	9

### Clinical outcome

Clinical follow-up included documentation of swelling, redness, joint effusion, delay of wound healing, crepitation and muscular atrophy. At no timepoint during the follow-up survey any differences could be determined between the two groups concerning these clinical parameters. Even though pain levels of the DePuy Synthes® VA-LCP group turned out to be higher in all of the follow-up examinations, significant differences could only be observed in the 6 weeks and 12 months follow-ups compared to the Medartis**®** Aptus Elbow group (see Table 2). Nerve injury had been detected in one patient with a partial sensomotoric palsy of the ulnar nerve, following ORIF of a comminuted AO type 13-C3 fracture using the DePuy Synthes® VA-LCP plating system (see Table 3). After neurolysis of the ulnar nerve the motoric palsy fully recovered whereas sensory ulnar nerve palsy remained.

**Table 2:** Functional elbow scoring was assessed using MEPs and QuickDash Scores for each plating system (DePuy Synthes® vs. Medartis**®** Aptus Elbow) separately, 6 weeks, 12 weeks, 6 months and 12 months postoperatively.

Scoring System	Plating System	6 weeks	12 weeks	6 months	12 months
VAS	DePuy Synthes® VA-LCP	Ø 4.7 +/− 1.1	Ø 1.4 +/−0.8	Ø 3.5 +/− 1.7	Ø 2.3 +/− 1.8
	Medartis**®** Aptus Elbow	Ø 2,3 +/− 2.2	Ø 0.8 +/− 0.9	Ø 1.1 +/− 1.5	Ø 1 +/− 0.9
	*p* value	0.006	0.163	0.630	0.074
Quick Dash	DePuy Synthes® VA-LCP	Ø 69.5 +/− 13.1	Ø 19.5 +/− 10.5	Ø 56.4 +/− 15.6	Ø 57.2 +/− 16
	Medartis**®** Aptus Elbow	Ø 74.1 +/− 21.1	Ø 52.3 +/− 4.8	Ø 25.5 +/− 21.2	Ø 25.5 +/− 10.1
	*p* value	0.634	0.092	0.002	0.002
MEPS	DePuy Synthes® VA-LCP	Ø 53 +/− 5.2	Ø 80 +/− 10	Ø 74 +/− 12.1	Ø 75.7 +/− 12.1
	Medartis**®** Aptus Elbow	Ø 75 +/− 10	Ø 91.4 +/− 12.1	Ø 90.6 +/− 10.7	Ø 90.9 +/− 10.7
	*p* value	0.001	0.009	0.004	0.007

Results are given as Ø mean +/− standard deviation

**Table 3:** Overview of the complications following fracture treatment for each plating system (DePuy Synthes® vs. Medartis**®** Aptus Elbow)

Complication	DePuy Synthes® VA-LCP	Medartis**®** Aptus Elbow
Non-union	0	2
Secondary fracture dislocation	1	0
Nerve injuries	1	0
Infections	0	0
Heterotopic ossifications	1	1

**Fig. 2:**
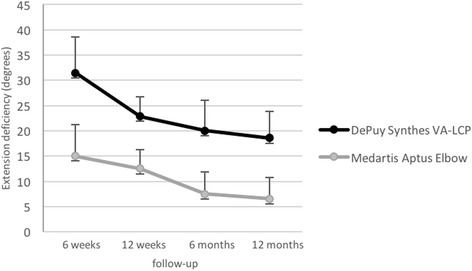
Illustration of the extension deficits. Comparison of extension deficits (Mean Value and Standard Deviation) following distal humeral plating (DePuy Synthes® vs. Medartis**®** Aptus Elbow) 6 weeks, 12 weeks, 6 months and 12 months postoperatively are presented in this figure. At all times of follow-up evaluation, the extension deficits showed significant differences between the two groups (*p* < 0.05)

Compared to the uninjured arm postoperative elbow extension deficiencies showed significant differences between both groups at every follow-up examination with a mean value of Ø 18 +/− 7.4 degrees in the DePuy Synthes® VA-LCP group compared to a mean value of Ø 6.5 +/− 7.5 degrees in the Medartis**®** Aptus Elbow group (*p* = 0.002) at 12 months postoperatively (see Fig. 2). For flexion and pronosupination no differences were seen between groups. Likewise, there were no signs of collateral ligament instability in both groups.

For subjective evaluation, patients rated their satisfaction for elbow use. Overall, 14 patients rated their satisfaction from very satisfied to sufficient (3× very satisfied, 6× satisfied, 4× moderate, 1× sufficient) and would undergo surgery again. The remaining three patients were not satisfied at time of last survey (2× Medartis**®** Aptus Elbow, 1× DePuy Synthes®). Differences between the two plating systems could not be detected for this subgroup analysis.

The elbow scoring showed a different result pattern for the QuickDash. In the first two clinical follow-ups (after 6 and 12 weeks) the DePuy Synthes® VA-LCP group showed better results than the Medartis**®** Aptus Elbow group, however not statistically significant. After 6 and 12 months on the other hand statistically significant better results were determined in the Medartis**®** Aptus Elbow group (see Table 2). The Medartis**®** Aptus Elbow group achieved significantly better MEP Scores in all of the postoperative follow-up examinations with good results 6 weeks postoperatively and excellent results 12 weeks, 6 months and 12 months postoperatively compared to the DePuy Synthes® VA-LCP group (see Table 2).

### Radiological follow-up

**Fig. 3:**
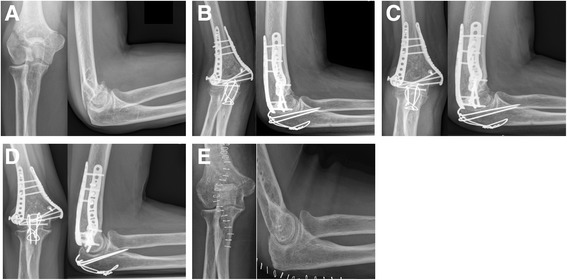
ORIF of a distal humerus fracture (AO 13-C3) with the Medartis® Aptus Elbow System. Radiographs of a 55-year-old female patient with a AO type 13-C3 fracture resulting from a bicycle accident: preoperative x-rays (**a**), postoperative results, 6 weeks (**b**), 12 weeks (**c**) and 6 months (**d**) following Medartis**®** Aptus Elbow plating. Implant removal could be obtained after fracture consolidation (**e**)

**Fig. 4:**
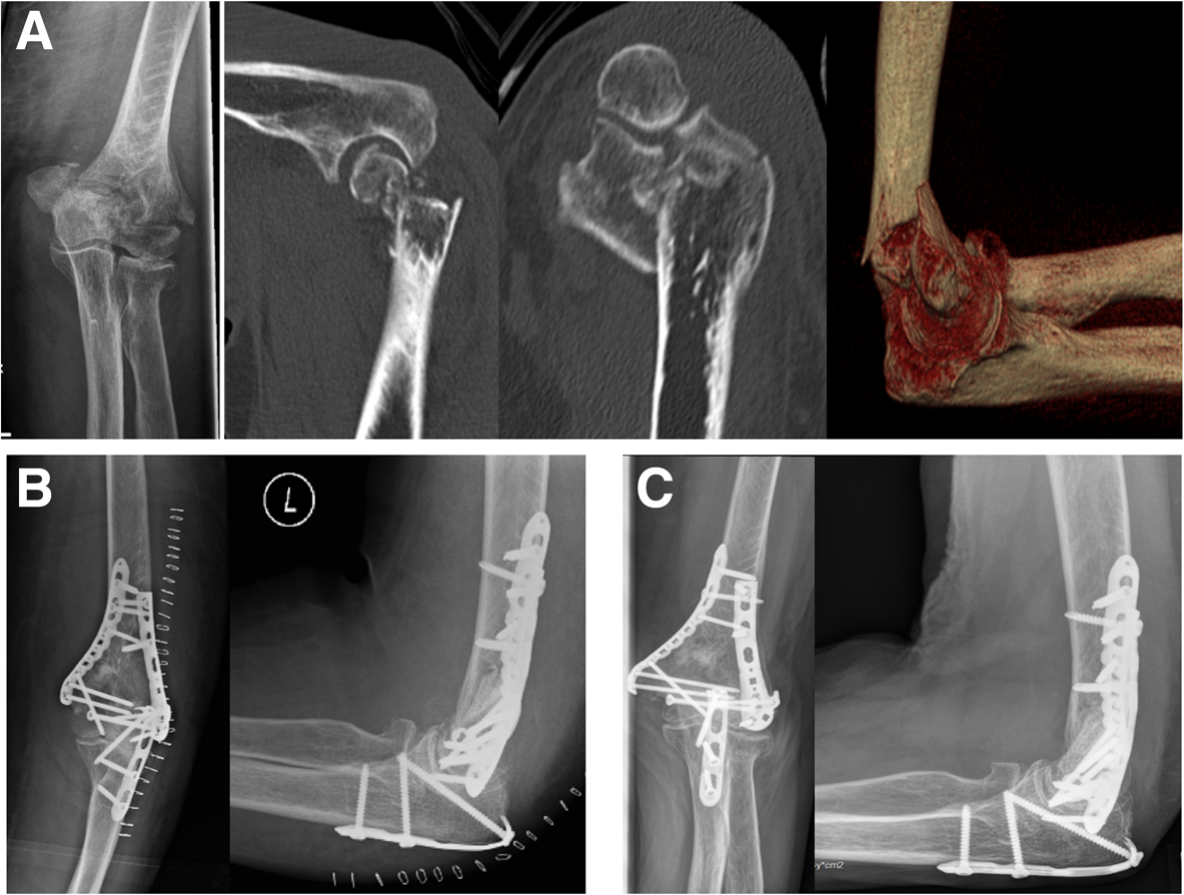
ORIF of a distal humerus fracture (AO 13-C3) with the DePuy Synthes® VA-LCP System. Radiographs and CT Scans of a 82-year-old female patient with a AO type 13-C3 fracture resulting from a fall. Preoperative x-rays and CT Scans (**a**), postoperative results, 6 weeks (**b**) and 12 months (**c**) following DePuy Synthes® VA-LCP plating

Eleven fractures showed a fracture healing without complications at final follow-up of 12 months (4× DePuy Synthes® vs. 7 x Medartis**®** Aptus Elbow) (see Figs. 3 and 4). Non-unions were detected in two patients, both following treatment of an AO type 13 C3 fracture with the Medartis**®** Aptus Elbow system. One of them, a 74-year-old female suffering from multiple comorbidities (i.e. diabetes type 2, obesity, COPD), presented with a non-union which resulted in secondary plate loosening. The other patient, 68-year-old female, presented as a heavy smoker (>25 pack years) and a history of recurrent falls of unknown cause, following initial distal humeral plating. Both patients have been revised using allogenic bone grafts with subsequent osseous healing. A secondary loss of reduction was seen in one 80-year-old female patient following treatment with the DePuy Synthes®, VA-LCP system. Due to her age and comorbidities, elbow replacement after plate removal was performed 5 months after index operation. Heterotopic ossifications were seen in two patients, one of each treatment group (see Table 3). One of them, a 55-years-old female (Medartis**®** Aptus Elbow), had to be revised due to ROM deficiencies based on massive heterotopic ossifications 13 months following plate osteosynthesis. In the sequel, an open arthrolysis and implant removal was performed after fracture consolidation. According to medical records, this patient was satisfied with the postoperative result 6 months after implant removal and arthrolysis.

## Discussion

A recent advance in the treatment of complex distal humeral fractures has been the introduction of anatomically preshaped, polyaxial angular stable locking plates [18–20]. Besides acting as internal fixators due to their locking fixation, screw positioning within a total range of up to 30° can be chosen, giving the surgeon a larger variety of screw placement [15]. The purpose of the present study therefore was to review the short-term results following treatment with these novel implants.

The most important finding was that usage of polyaxial angular stable plating systems leads to reliable results with good functional outcome in the majority of patients. Substantial differences between both evaluated plating systems could neither be detected for subjective evaluation nor for functional elbow scoring. Patients’ satisfaction is directly constrained to elbow mobility. The postoperative ROM showed significant better results with decreased extension deficits in the Medartis**®** Aptus Elbow group compared to the DePuy Synthes® group (see Fig. 2). However, an overall extension deficit of 12.3° (see Fig. 2) 12 months after osteosynthesis was found for both groups which is sufficient to fulfil most of the personal daily routine activities (functional arc of elbow motion according to BF Morrey) [10]. While both plating systems provide comparable implants in terms of diameter, length and material, there might be advantages in the plate design of the lateral Medartis**®** Aptus Elbow plates, which are twisted from distal lateral to proximal posterior for parallel plating in order to reduce soft tissue detachment during the operation and postoperative soft tissue irritation as well. This design feature might at least explain parts of the slightly higher postoperative ROM, even though not proven based on the present data. Obvious restrictions in the postoperative ROM or postoperative stiffness, however, defy explanation by implant size or configuration. Rather reasonable for postoperative stiffness in general, is the prolonged postoperative immobilization, often used to compensate inadequate fixation [21]. As another reason for postoperative stiffness, heterotopic ossifications or capsule adhesions are described with rates up to 8.6% in the current literature [22]. Two patients with extension deficiencies due to heterotopic ossifications were determined in our trial (see Table 3), with only one of them needing revision after fracture consolidation. In cases of major extension deficiencies open arthrolysis and implant removal is recommended [22], as we performed in our case with good clinical outcome. Postoperative Indomethacin therapy seems promising to prevent from heterotopic ossifications following ORIF of distal humeral fractures, though significant advantages are still missing in current literature [22]. In this survey no postoperative prophylaxis for heterotopic ossifications was performed. However, different plate configurations are not known to have an impact on postoperative heterotopic ossifications. Known risk factures are rather associated brain injury or delayed surgical treatment [22].

Knowing that bicolumnar plating is an effective and reliable method in treating complex distal humeral fractures [6, 23], there is little evidence of the ideal plate positioning [24, 25]. Especially the differences of orthogonal plating vs. parallel plating are discussed controversially in current literature. While few authors showed higher biomechanical stability following orthogonal (90°-90°) plating, especially in the osteoporotic bone [23, 26], other authors refer to higher stability rates following parallel plaiting [21, 27, 28]. Current biomechanical examinations of Kudo et al. [29], using the DePuy Synthes® VA-LCP system showed a higher rate of secondary displacement in the orthogonal plating orientation than in the parallel orientation. In matters of theses controversial studies we believe, according to the latest publication of Govindasamy et al. [25], that plate orientation should be chosen according to the fracture pattern, as both variants seem to provide enough stability to fix distal humerus fractures.

Besides the previously discussed plate positioning, screw orientation is known to influence fracture stability, with conventional angle stable locking systems providing higher stability especially against rotational failure than variable locking systems [30]. Highest stability in variable angle locking systems can be achieved inserting the screws perpendicular to the plate [30]. So, a higher variability in fragment fixation using variable plating systems, seems to be at the expense of stability. However, statistically significant differences in postoperative stability due to the used two different implant systems, with only one patient showing a secondary loss of reduction following treatment with the DePuy Synthes® VA-LCP and orthogonal plating position could not be detected in our study.

The complication rates in our study (see Table 3) are consistent with the rates described in the current literature, accounting up to 35% [19]. These high rates can be explained due to numerous reasons, such as the complexity of distal humerus fractures, the low amount of cancellous bone in the distal fragments and the tendency of the elbow joint to develop joint stiffness [10, 19, 31].

This study has several limitations, including the small number of only 17 examined patients at last follow-up survey and the disparity of both groups (7 patients DePuy Synthes® VA-LCP vs. 10 patients Medartis**®** Aptus Elbow). Due to the small number of patients, a differentiation between the treated fracture types and the used plate positioning (parallel plating vs. perpendicular plating) was not reasonable with respect to functional outcomes. Also, the influence of implant removal was not analysed. However, the presented follow-up rate of 85%, the wide assessment of functional parameters and the prospective randomized character certainly present the strengths of the present survey. To the best of our knowledge, moreover, this is the first randomized controlled trial to compare two modern polyaxial plating systems in distal humeral fracture treatment.

## Conclusion

The usage of anatomically preshaped low profile locking plates in fracture treatment of distal humeral fractures leads to good clinical results. Even though there might be a trend of some advances of the Medartis**®** Aptus Elbow plating system concerning postoperative ROM and elbow scoring, a crucial difference in the overall clinical outcome between the two plating systems could not be detected in the short-term follow-up. Further trials with higher sample sizes are needed to prove the advantages of these novel implant systems in the treatment of these challenging fractures.

To sum up, successful management of distal humerus fractures is essentially constrained to a correct fracture reduction, a sufficient reconstruction of the articular surface and a high primary stability for early functional rehabilitation.

## Abbreviations

MEPS: Mayo elbow performance score
ORIF: Open reduction internal fixation
QuickDASH: Disabilities of the arm, shoulder and hand score
ROM: Range of motion
VAS: Visual analogue scale
